# An Educational Bioinformatics Project to Improve Genome Annotation

**DOI:** 10.3389/fmicb.2020.577497

**Published:** 2020-12-07

**Authors:** Zoie Amatore, Susan Gunn, Laura K. Harris

**Affiliations:** ^1^Science Department, Harris Interdisciplinary Research, Davenport University, Lansing, MI, United States; ^2^College of Urban Education, Davenport University, Grand Rapids, MI, United States

**Keywords:** bioinformatics, hypothetical protein, genome annotation, education, classroom, undergraduate

## Abstract

Scientific advancement is hindered without proper genome annotation because biologists lack a complete understanding of cellular protein functions. In bacterial cells, hypothetical proteins (HPs) are open reading frames with unknown functions. HPs result from either an outdated database or insufficient experimental evidence (*i.e.*, indeterminate annotation). While automated annotation reviews help keep genome annotation up to date, often manual reviews are needed to verify proper annotation. Students can provide the manual review necessary to improve genome annotation. This paper outlines an innovative classroom project that determines if HPs have outdated or indeterminate annotation. The Hypothetical Protein Characterization Project uses multiple well-documented, freely available, web-based, bioinformatics resources that analyze an amino acid sequence to (1) detect sequence similarities to other proteins, (2) identify domains, (3) predict tertiary structure including active site characterization and potential binding ligands, and (4) determine cellular location. Enough evidence can be generated from these analyses to support re-annotation of HPs or prioritize HPs for experimental examinations such as structural determination via X-ray crystallography. Additionally, this paper details several approaches for selecting HPs to characterize using the Hypothetical Protein Characterization Project. These approaches include student- and instructor-directed random selection, selection using differential gene expression from mRNA expression data, and selection based on phylogenetic relations. This paper also provides additional resources to support instructional use of the Hypothetical Protein Characterization Project, such as example assignment instructions with grading rubrics, links to training videos in YouTube, and several step-by-step example projects to demonstrate and interpret the range of achievable results that students might encounter. Educational use of the Hypothetical Protein Characterization Project provides students with an opportunity to learn and apply knowledge of bioinformatic programs to address scientific questions. The project is highly customizable in that HP selection and analysis can be specifically formulated based on the scope and purpose of each student’s investigations. Programs used for HP analysis can be easily adapted to course learning objectives. The project can be used in both online and in-seat instruction for a wide variety of undergraduate and graduate classes as well as undergraduate capstone, honor’s, and experiential learning projects.

## Introduction

Nucleic acid sequencing has become so inexpensive that researchers are generating a plethora of fully sequenced genomes annually through massive initiatives such as the Earth BioGenome Project which aims to sequence the genomes of 1.5 million eukaryotic species by 2050 (Yandell and Ence, 2012; Lewin et al., 2018). Once a genome sequence is determined, it must be annotated to identify the locations and functions of genes (Koonin and Galperin, 2003). In bacteria, the first step in genome annotation is identifying open reading frames (ORFs). An ORF is a continuous stretch of DNA that begins with a start codon and ends at a stop codon and has the proper number of nucleotides to potentially encode a functional protein (Brown, 2002). Due to the lack of introns and exons in bacterial genes, an ORF is usually synonymous with a gene in bacteriology. The amino acid (*i.e.*, primary protein) sequence for each ORF is used to search several databases to predict gene function. These databases include (1) sequence databases to identify sequence similarities with established sequences, (2) domain databases to detect conserved domains, (3) genome-oriented databases for identification of orthologous relationships for refined functional prediction, and/or (4) metabolic databases for metabolic pathway reconstruction (Koonin and Galperin, 2003). From these data, a public knowledgebase record for each ORF is generated which typically includes nucleic acid and amino acid sequences, gene and protein sizes, any identified domains, and a predicted function. The record is easily retrievable via a unique identifier (*i.e.*, locus tag) which is consistently used across knowledgebases (Brown et al., 2015; Tatusova et al., 2016; Coordinators, 2018). These public records are used for a wide variety of gene analyses, such as pathway enrichment, so having proper genome annotation is important to draw accurate and complete scientific conclusions (Goad and Harris, 2018; Smits, 2019).

Unfortunately, many genomes have a substantial number (up to 70%) of hypothetical proteins (HPs), which are ORFs with unknown functions (Sivashankari and Shanmughavel, 2006; Mohan and Venugopal, 2012; Bharat Siva Varma et al., 2015; Ijaq et al., 2015; Islam et al., 2015; School et al., 2016). Reports estimated that around 33% of National Center for Biotechnology Information (NCBI) knowledgebase sequences in 2006 were HPs (Kolker et al., 2004; Sivashankari and Shanmughavel, 2006; Omeershffudin and Kumar, 2019). While the exact number of HPs in today’s NCBI is unknown, recent papers on *Mycobacterium tuberculosis* and *Exiguobacterium antarcticum* strain B7 genomes report around 27% HPs (da Costa et al., 2018; Yang et al., 2019) with 16% HPs in *Shigella flexneri* (Gazi et al., 2018). Assuming 20% of the current 218,642,238 GenBank sequences are HPs, over 43 million proteins need proper annotation, and this number continues to grow exponentially as sequences continue to be deposited. A hypothetical protein (HP) can be the result of either outdated or indeterminate annotation. Outdated HPs result from an out-of-date knowledgebase. Older genomes are more likely to have outdated HPs since experimental work to determine function of HPs is ongoing and annotation for older genomes was completed prior to the characterization of a similar sequence with known function. Automated and manual curation of public knowledgebases is needed to improve genome annotation and identify sequences with out-of-date annotation. For example, function was successfully attributed to approximately 17% of HPs in *E. antarcticum* strain B7 using computational methods (da Costa et al., 2018). If computational approaches can re-annotate just 10% of current HPs, then annotation will be improved for over 4 million proteins, which would substantially improve public knowledgebases overall. Alternatively, indeterminate annotation is the result of true HPs whose amino acid sequence has low similarity to proteins with known function. Experimental work is needed to properly annotate true HPs and improve genome annotation, but once completed manual inspection is needed to further discover, analyze, and correct erroneous annotation.

**FIGURE 1:**
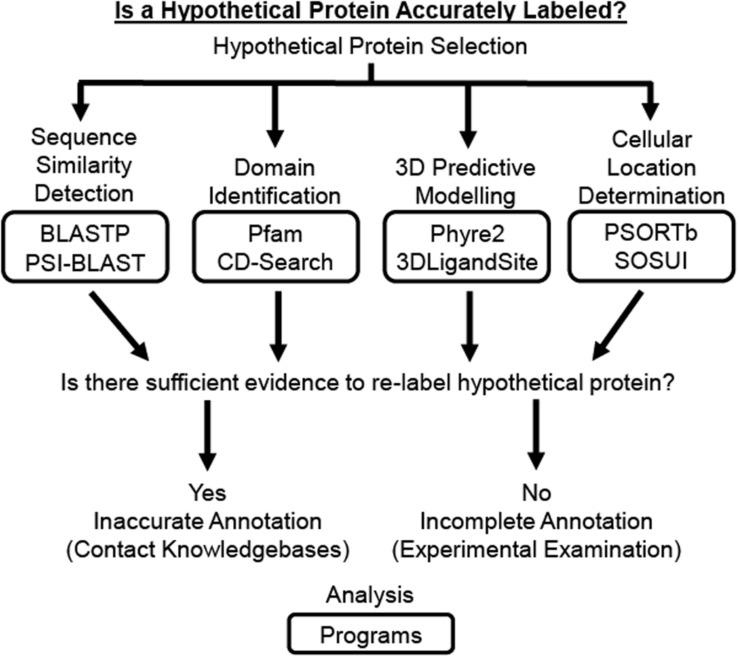
Schematic of Hypothetical Protein Characterization Project. The Hypothetical Protein Characterization Project provides students with a process that generates evidence to address if a hypothetical protein (HP) is accurately labeled. The HP can be selected randomly, through differential gene expression analysis using established statistical methods, or phylogenetic relations established through sequence similarity. Once selected, the HP’s amino acid sequence is analyzed by web-accessible individual programs for (1) detection of sequence similarities, (2) identification of protein domains, (3) 3D predictive modeling of the HP’s structure including active site and potential ligand binding partners, and (4) determination of protein cellular location. If results from these analyses provide sufficient evidence to support a function for the HP, the results can be provided directly to knowledgebases so the protein’s public record can be updated. Otherwise, the HP needs experimental examination before a function could be assigned.

Several previously reported studies have used computational approaches to assign functional annotation to HPs in a wide range of bacterial and viral species, including but not limited to *Staphylococcus aureus* (Mohan and Venugopal, 2012; School et al., 2016), *M. tuberculosis* (Raj et al., 2017; Yang et al., 2019), *Vibrio cholerae* (Islam et al., 2015), *Klebsiella pneumoniae* (Pranavathiyani et al., 2020), *Mycoplasma pneumoniae* (Shahbaaz et al., 2015), *Orientia tsutsugamushi* (Imam et al., 2019), *Corynebacterium pseudotuberculosis* (Araujo et al., 2020), human adenovirus (Dorden and Mahadevan, 2015; Naveed et al., 2017), and vaccinia virus (Mahmood et al., 2016). These studies utilize some combination of the various computational tools and databases available to analyze the physiochemical, functional, and structural properties of an HP (Table 1) since results generated from a single server cannot provide a complete functional determination currently (Dorden and Mahadevan, 2015). While these computational resources are continually changing, due to their wide application in research it would be beneficial for undergraduate microbiology students to be familiar using some of the more enduring and commonly referenced resources. Therefore, this paper introduces a Hypothetical Protein Characterization Project based off commonly referenced resources in previously reported *in silico* HP characterization studies that students use while learning interdisciplinary concepts in bioinformatics, microbiology, biochemistry, and genetics (Figure 1). This educational, inquiry-based bioinformatics project familiarizes students with multiple free web-accessible programs that identify and predict HP characteristics, such as sequence similarities to other proteins, protein domains, tertiary (*i.e.*, 3D) protein structure, ligand binding partners, and cellular location. Critical thinking skills applied by the student to results obtained from the Hypothetical Protein Characterization Project are used to determine whether an HP has outdated or indeterminate annotation. This determination can be useful for improving public knowledgebase annotation and prioritizing experimental examination of true HPs.

**TABLE 1:** Example studies considered in the development of the Hypothetical Protein Characterization Project.

**Species**	**Citation**	**No. HPs**	**Resources Used**
*Staphylococcus aureus*	Mohan and Venugopal, 2012	10	CDD-BLAST, Pfam, PS^2^, STRING, QFinder, ExPASy ProtParam, SOSUI, DISULFIND
	School et al., 2016	35	PSI-BLAST, ExPASy ProtParam, CDD-BLAST, Pfam, PS^2^, 3DLigandSite, STITCH, STRING, PSORTb, SOSUI, DISULFIND
*Mycoplasma pneumoniae*	Shahbaaz et al., 2015	204 (41%)	BLAST, FASTA, HMMER, SBASE, CATH, SUPERFAMILY, InterPro, SYSTERS, CDART, SMART, GPCRpred, Discovery Studio, STITCH, STRING, iPfam, ExPASy ProtParam, PSORTb, PSLpred, LOCTree3, TMHMM, HMMTOP, SignalP 4.1, SecretomeP, VirulentPred, DBETH server
*Mycobacterium tuberculosis*	Raj et al., 2017	1055 (55%)	BLASTP, ExPASy ProtParam, PSORTb, CELLO, TMHMM, SignalP 4.1, HHPred, HMMSCAN, Pfam, InterPro, SUPERFAMILY, VirulentPred, VICMPred
*Klebsiella pneumoniae*	Pranavathiyani et al., 2020	540	InterPro, Pfam, BLASTP, CELLO2GO, GO FEAT, STRING, ExPASy ProtParam, VICMpred, MP3, I-TASSER
*Corynebacterium pseudotuberculosis*	Araujo et al., 2020	172 (47%)	GO FEAT, Pfam, CATH, SUPERFAMILY, VICMPred, CDART, CDD-BLAST, ExPASy ProtParam, PSORTb, TopHat, Gipsy, VirlentPred, STRING, PSIPRED, Modeler
*Vibrio cholerae*	Islam et al., 2015	6	CDD-BLAST, Pfam, PS^2^, STRING, QFinder, ExPASy ProtParam, PSORTb, DISULFIND
*Orientia tsutsugamushi*	Imam et al., 2019	344	BLASTP, ExPASy ProtParam, PSLpred, CELLO, ScanProsite, SMART, Motif Scan, PFP-FunDSeqE, VirulentPred, PFP, Argot2, PSIPred, Modeler
Vaccinia virus	Mahmood et al., 2016	1 (100%)	BLAST, GOR IV server, I-TASSER, ExPASy ProtParam PSI-BLAST and Clustal Omega used to select model template for I-TASSER
Human adenovirus	Dorden and Mahadevan, 2015	28	BLASTP, Pfam, SMART, Phyre2, SWISS-MODEL, MuFOLD, PFP, ESG, Argot2, BAR+, PSIPred, ProtFun, dcGO, 3d2GO
	Naveed et al., 2017	38 (16%)	BLASTP, Pfam, CATH, SUPERFAMILY, INETRPRO, MOTIF, CDART, SMART, SVMPort, ProtoNet, I-TASSER, ExPASy ProtParam, Virus PLoc, TMHMM, HMMTOP, DISULFIND

*No. HPs, Total number of hypothetical proteins examined (percent of hypothetical proteins with proposed annotation revisions if available).*

## Hypothetical Protein Selection

The first step in the Hypothetical Protein Characterization Project is the selection of HPs to be characterized. This section details three general approaches for HP selection (Table 2). HPs can be selected randomly or targeted through differential gene expression analysis or phylogenetic relations.

**TABLE 2:** Selected approaches for hypothetical protein selection.

**Approach**	**Sub-approach**	**Description**	**Level^1^**	**Setting(s)^2^**
Random	Student-directed	Complete student autonomy to select HPs for characterization	Beginner	C
	Instructor-directed	Instructors limit student ability to select HPs for characterization (*e.g.*, students select HPs from genome of “class pet microbe”)	Beginner	C
Differential Gene Expression	Single-gene Analysis	Use of statistical method(s) (*e.g.*, *T*-test and/or fold change) on gene expression data to find and prioritize individual differentially expressed HPs for characterization	Intermediate	C, E, H, G
	Singular Enrichment Analysis	Gene enrichment analysis comparing groups of significant HPs with similar differentially expression as defined by single-gene analysis	Intermediate	C, E, H, G
	Gene Set Enrichment Analysis	Gene enrichment analysis comparing a group of the most differentially expressed HPs to a gene signature (*i.e.*, gene list ranked by differential expression based on a statistical method)	Advanced	E, H, G
Phylogenetic Relations	N/A	HPs for characterization are selected for their sequence similarities to proteins with established tertiary structures	Intermediate	E, H, G

*^1^Level definitions: Beginner, does not require additional steps or prior knowledge of statistics; Intermediate, may require prior knowledge of statistics and/or additional steps using free web-accessible programs; Advanced, requires prior knowledge of statistics and additional steps using publicly available free downloadable programs. ^2^C, classroom; E, experiential learning courses; G, graduate projects; H, undergraduate honors and capstone projects. HPs, hypothetical proteins.*

### Random Selection

Depending on instructor preference and learning objectives, students can be allowed to select HPs themselves (*i.e.*, student-directed) or selection can be partially or completely directed by the instructor (*i.e.*, instructor-directed). Students can find HPs easily by searching the NCBI knowledgebase for the term “hypothetical protein” to generate a list for selection, as done previously (Bharat Siva Varma et al., 2015). Further, if the student is interested in a specific organism, HPs can be selected randomly using NCBI’s Genome database.

Alternatively, instructors may choose to partially or completely direct HP selection. One way a project can be partially instructor-directed is by requiring the class to designate a class pet microbe. The instructor then provides a list of available HPs from the class-appointed pet microbe for student selection. The class pet microbe technique is based on early published computational characterization studies that limited focus to HPs that were randomly selected from several hundred HPs in one highly pathogenic bacterial species (Mohan and Venugopal, 2012; School et al., 2016). To reduce the number of potential HPs for selection, a protein size cut-off can be imposed also (Shahbaaz et al., 2015).

### Differential Gene Expression

The differential gene expression approach requires gene expression data, such as those produced by microarray or RNAseq procedures, containing at least two groups (*i.e.*, experimental and control) that are useful for comparison. HPs that have the greatest change in gene expression between groups (*i.e.*, differential gene expression) are given the highest priority for HP selection. Gene expression datasets that measure expression for nucleotide sequences associated with HPs can be generated by the student in the laboratory or found in the Gene Expression Omnibus (GEO) database (Edgar et al., 2002; Barrett et al., 2011, 2013).

**FIGURE 2:**
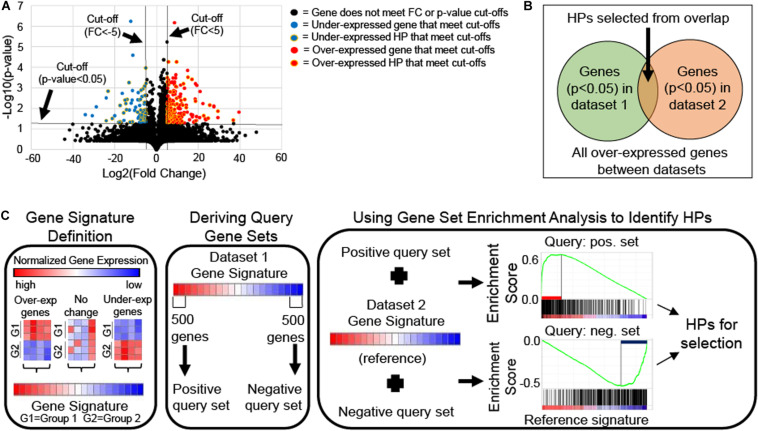
Schematics of differential gene expression approaches for hypothetical protein (HP) selection. **(A)** Volcano plot of mRNA expression data from Gene Expression Omnibus accession number GSE46687 identified HPs with statistical (two-tailed Welch’s *T*-test *p*-value < 0.05) and biological relevance [fold change (FC) > 5 for over-expressed or <–5 for under-expressed genes in experimental compared to control groups] to antibiotic resistance in *Staphylococcus aureus* that could be selected for the Hypothetical Protein Characterization Project. **(B)** Venn diagram illustrates conceptually how HPs are selected from singular enrichment analysis using the overlap of statistically significant (e.g., *T*-test *p*-value < 0.05) over-expressed genes between two mRNA expression datasets. The same concept applies to selecting under-expressed HPs also. **(C)** Schematic shows how HPs can be selected from gene signature comparison using Gene Set Enrichment Analysis (GSEA). Gene signatures are gene lists ranked by their differential expression based single-gene analysis (e.g., T-score or FC). A gene signature for each of two mRNA expression datasets are generated. One signature is chosen from which the 500 most over- and under-expressed genes are taken to derive positive and negative query gene sets, respectively. Each query gene set is compared individually to the second gene signature, which is used as reference for GSEA. GSEA calculates an enrichment plot with a maximum enrichment score. GSEA identifies leading-edge genes, which are genes that contribute most to reaching the maximum enrichment score. HPs among leading-edge genes are selected for the Hypothetical Protein Characterization Project.

If only two groups are available, HPs can be selected using single-gene analysis approach which requires meeting a statistical cut-off, like a *T*-test *p*-value <0.05. This approach can produce long lists of differentially expressed HPs that may contain redundancy and cannot be prioritized based on biological relevance, thus prioritization of HPs for characterization, require utilization of statistical methods. For example, volcano plots (*i.e.*, scatter plot that compares a gene’s statistical significance via *T*-test *p*-value to its biological relevance via fold change) are frequently used to identify differentially expressed genes (Li, 2012; Kumar et al., 2018). Differentially expressed HPs with the best statistical significance (*i.e.*, lowest *p*-value) and biological relevance (*i.e.*, highest fold change) are given selection priority for the Hypothetical Protein Characterization Project (Figure 2A).

If more than two experimental groups are available, HPs can be selected by gene enrichment analysis (Goad and Harris, 2018). HPs can be selected by either singular enrichment analysis or gene set enrichment analysis (Huang et al., 2009; Tipney and Hunter, 2010). In singular enrichment analysis, each gene is considered individually via single-gene analysis, generating multiple lists of statistically significant HPs, one from the differential expression comparison of each experimental group relative to the control. HP lists are then examined for overlapping HPs, which are considered most relevant to the phenotypic variation under examination (Figure 2B).

Alternatively, gene set enrichment analysis (GSEA) compares gene signatures (*i.e.*, list of genes ranked by their differential expression based on an appropriate statistic method such as *T*-test or fold change) rather than individual genes. To do this, one gene signature is used as reference (*i.e.*, all genes are used) and the other signature is used to generate two separate query gene sets derived from the signature’s positive and negative tails (*i.e.*, representing the most over-or under-expressed genes in the gene signature, respectively). Query gene sets must include between 15 and 500 genes for GSEA to properly function (Subramanian et al., 2005), and to maximize potential HPs for selection we recommend using a 500 gene inclusion size. GSEA compares the reference signature to each query gene set individually to calculate an enrichment score (Figure 2C). Genes that contribute most to reaching the maximum enrichment score for GSEA are called leading-edge genes and are thought to contribute to the phenotypic difference under examination. HPs included among identified leading-edge genes are given the highest priority in HP selection. GSEA requires use of specialized software with a JAVA-based, user-friendly desktop version freely available at Broad Institute (Subramanian et al., 2005).

### Sequence Similarity to a Protein With Determined Structure

The sequence similarity to a protein with determined structure approach can find outdated HPs for characterization, as we demonstrate in section 4.1. To select HPs using this approach, students begin by finding established proteins that have already undergone some experimental examination, such as protein structure determination via X-ray Crystallography, and therefore have accurate annotation. The Research Collaboratory for Structural Bioinformatics (RCSB) Protein Data Bank (PDB) is a rich resource for finding established proteins since it is the largest free and publicly available archives of macromolecular structural data (Bank, 1971; Berman et al., 2000, 2014; Burley et al., 2017). Next, amino acid sequences from established proteins undergo sequence similarity searches using programs such as the Position-Specific Iterated Basic Local Alignment Search Tool (PSI-BLAST) to select HPs for the Hypothetical Protein Characterization Project.

## Analysis of Hypothetical Proteins

After an HP is selected for characterization, the amino acid sequence in FASTA format is acquired from a public knowledgebase like NCBI or UniProt, and used to detect sequence similarities, identify protein domains, predict protein tertiary structure including active site and potential ligand binding partners, and determine cellular location (Table 3). Instructional videos for use of each program discussed in this section are available on our “Online Faculty Mentoring Network to Develop Video Tutorials” YouTube channel^1^.

**TABLE 3:** Selected analysis programs for Hypothetical Protein Characterization Project.

**Objective**	**Program**	**Citation**	**Description**
Sequence Similarity Detection	BLASTP	Altschul et al., 1990	Encompasses similarities between relevant sequences to predict the functionality and evolutionary aspect of sequences between gene families.
	PSI-BLAST	Altschul et al., 1997; Altschul and Koonin, 1998	Provides means of detection to note distant relationships between proteins.
Domain Identification	Pfam	Sonnhammer et al., 1998; El-Gebali et al., 2019	Database of functional proteins that are called domains. Provides the students with structure of the protein, family annotation, and protein search against database models.
	CD-Search	Marchler-Bauer and Bryant, 2004; Lu et al., 2020	Protein annotation that contains annotated sequence alignment models along with complete proteins. The output allows for identification of domains in the form of matrices.
3D Predictive Modeling	PHYRE2	Kelley et al., 2015	Provides affiliation of proteins to predict protein structure, function, and mutation. Software uses a detection method through homologs to build 3D models, note binding sites, and analyze amino acids.
	3DLigandSite	Wass et al., 2010	Allows for the prediction of ligand binding sites by using the predicted protein structure.
Cellular Location Determination	SOSUI	Hirokawa et al., 1998	Provides transmembrane domain prediction of a single alpha helix. This process occurs through scanning through protein sequence to identify hydrophobic regions.
	PSORTb	Yu et al., 2010	Contains multiple modules to analyze biological features of known characteristics pertaining to subcellular localization. Thus, the database may predict a protein localization site. Database also encompasses Gram-negative and Gram-positive localization features.

### Sequence Similarity Detection

Detecting sequences that share significant similarity to an HP is an important first step in analysis since similar sequences are thought to be homologous and likely share a common ancestor (Pearson, 2013). Widely used similarity search programs, like the Basic Local Alignment Search Tool (BLAST), are used to estimate similarity between sequences (Altschul et al., 1990). Results from any BLAST program includes the percentage of query (*e.g.*, amino acid) coverage and identity to individual sequences, with high percentages of query coverage and identify to sequences with known function indicating an outdated HP. Further, a bit-score indicates the required size of the database needed to find the same sequence similarity by random chance with a high bit-score indicating sequence similarity. To estimate the statistical significance of detected similarities, the bit-score is used to calculate an Expect-value (*E*-value), representing the number of closely matched sequences that are anticipated by random change when searching a database of certain size (*i.e.*, random background noise). *E*-values close to zero highlight similar sequences.

At NCBI’s website there are several BLAST programs available for use. Nucleotide BLAST (BLASTN) and Protein BLAST (BLASTP) detect sequence similarities between other nucleotide and amino acid sequences, respectively. While either BLAST program can be used and comparing between BLASTN and BLASTP would generate a good educational discussion, the Hypothetical Protein Characterization Project uses BLASTP to reduce student confusion by providing input consistency across HP analysis. The Hypothetical Protein Characterization Project also looks at results from Position-Specific Iterated BLAST (PSI-BLAST). PSI-BLAST first generates the same results as BLASTP sequence alignments to establish a specialized position-specific scoring matrix (PSSM) from all user-selected sequences, representing what the group of sequences might look like on a positional basis. Use of PSSM allows for the comparison of local amino acid sequence patterns between proteins rather than direct comparison of amino acid sequences themselves. Therefore, through several rounds of computational analysis (*i.e.*, iterations), PSI-BLAST refines the PSSM for an HP based on PSSM alignments with user-selected sequences identified within each iteration. This process combines underlying conservation information from a range of related sequence into a single score matrix (Altschul et al., 1997; Bhagwat and Aravind, 2007). By using this PSSM methodology, PSI-BLAST can detect less similar sequences and is more likely to identify HPs. True HPs, by definition, cannot have similar sequences with established function. Thus, identification of similar sequences with known function using BLAST can strongly indicate outdated annotation for the HP being analyzed.

### Domain Identification

Protein domains are spatially distinct and compact regions of a protein that can fold into a stable structure that may be integral to the protein’s function (Yegambaram et al., 2013). Domains are often conserved across proteins with similar function across diverse species. There are several protein domains databases that are readily available. For example, the Pfam database has been collecting protein information since 1995 and now contains more than 17,000 entries (Sammut et al., 2008; Finn et al., 2010; El-Gebali et al., 2019; Lu et al., 2020). Pfam has a large collection of protein domains, which are individually represented by hidden Markov model (HMM) based profiles and multiple sequence alignments (Sonnhammer et al., 1998). While Pfam is a trusted resource, it can be expanded upon. NCBI’s Conserved Domain Database (CDD) is a collection of multiple sequence alignment models for full-length proteins and ancient domains that includes NCBI-curated domains, which use 3D-structure information to define domains, and domain models imported from several external databases including Pfam (Lu et al., 2020). The CDD can be searched using the CD-Search tool which is easily accessible from NCBI’s Protein Database. Conserved domain (CD)-Search uses RPS-BLAST, a PSI-BLAST variant, to scan a protein for any sets of pre-calculated position-specific scoring matrices (Marchler-Bauer and Bryant, 2004). CD-Search results are presented as an annotation of protein domains with high confidence associations. These associations are determined by calculating the *E*-value between the protein’s sequence and any domains are shown as specific hits using similar methods to those previously described for BLAST programs. The Structural Classification of Proteins (SCOP) database of proteins with known structures that organizes protein domains by their evolutionary and structural relationships, providing a broad overview of established protein folds, detailed information about any close relatives to an HP, and a general framework for future protein classification (Andreeva et al., 2014, 2020). SUPERFAMILY is a database of structural and functional protein annotation based on a collection of HMMs representing SCOP superfamily structural domains (Gough et al., 2001). The Conserved Domain Architecture Retrieval Tool (CDART) and Simple Modular Architecture Research Tool (SMART) can be used to identify similarities across significant evolutionary distances through comparing domain architecture (*i.e.*, sequential order of conserved domains in a protein sequence) for protein (Geer et al., 2002)and genetically mobile domains (Schultz et al., 1998; Letunic and Bork, 2018), respectively, both using PSI-BLAST. Further, the CATH protein domain database classifies protein secondary structures from the PDB and collects domains into superfamilies only when there is enough evidence of divergence from a common ancestor (Sillitoe et al., 2019). The CATH database is paired with Gene3D which uses CATH’s information to predict structural domain locations for protein sequences available in public databases, allowing for functional information and active site residue annotations (Lewis et al., 2018). Since domains are distinct regions of a protein, it is not uncommon for a protein to have more than one identified domain, ergo results from searching these domain databases also usually identify the range of amino acids associated with domains of HPs under investigation. HPs containing at least one domain with an established function likely have outdated annotation.

### 3D Predictive Modeling

3D predictive modeling gives students the ability to consider an HP’s tertiary structure and potential binding partners. To do this, the Structural Bioinformatics Group at Imperial College London developed a suite of integrative modeling programs, Protein Homology/analogY Recognition Engine V 2.0 (Phyre2), with free web portal access (Kelley et al., 2015). Phyre2 uses template-based modeling (*i.e.*, homology and comparative modeling) based on a three-step procedure. First, homologous sequences are gathered by scanning a query sequence against specially curated protein sequence database with HHblits. This produces a multiple-sequence alignment that is used by PSIPRED to predict secondary structure before both the alignment and secondary structure prediction combined into a query HMM. Next, the query model is scanned against a database of HMMs of proteins of known structure. From this search, top-scoring alignments are used to generate an unrefined backbone-only model. Finally, the model is refined via loop modeling and side-chain placement. Template-based modeling as used by Phyre2 is a good approach assuming homology exists between a user-supplied sequence and at least one sequence of known structure, meaning Phyre2 and any other template-based modeling programs are unable to model true HPs. If the Phyre2 generated model is assigned a >90% confidence and does not contain substantial disorder (<50%), Phyre2 automatically submits the model and its corresponding amino acid sequence to the 3DLigandSite server for ligand binding site prediction (Wass et al., 2010). In a similar approach to template-based modeling, 3DLigandSite identifies structures like the one generated by Phyre2 model and superimposes bound ligands from identified structures onto the model. This is done multiple times to establish a cluster of the highest number of ligands for active site prediction. It may take several hours for Phyre2 and 3DLigandSite to generate results, however, those results include: (1) tables of identified ligand clusters and binding-site residues, (2) visual representations of the model, and (3) predicted binding site and any ligand clusters. Thus, 3D predictive modeling can identify outdated HPs due to theoretical tertiary structure homologies with proteins of known function.

There are several other computational resources available to predict an HP’s tertiary structure from its primary (*i.e.*, amino acid) sequence and predict its potential binding partners. Alternatives to Phyre2 include but are not limited to SWISS-MODEL (Schwede et al., 2003; Waterhouse et al., 2018), PS^2^ (Chen et al., 2006, 2009), and the Iterative Threading Assembly Refinement (I-TASSER) program (Roy et al., 2010; Yang and Zhang, 2015). SWISS-MODEL is the original fully automated protein homology modeling server. In its most recent version, SWISS-MODEL uses a ProMod3 that differs from prior versions and other programs like Phrye2 by replacing *ab-initio* techniques to resolve insertions and deletions in the aligned template structure with structural database searches for viable candidate fragments. PS^2^ is another automatic homology modeling server that uses a substitution matrix, S2A2, to combine sequence and secondary structure information to detect established proteins with remote similarity before the 3D structure is generated via the MODELER modeling package (Sali and Blundell, 1993; Webb and Sali, 2014). MODELER uses an alignment between the HP’s sequence and known related structures to generate a model containing all non-hydrogen atoms based on satisfying atomic spatial restraints. The I-TASSER is an integrated platform for automated protein structure and function prediction from an amino acid sequence that is based on a sequence-to-structure-to-function paradigm. To accomplish this, I-TASSER begins by using multiple threading alignments and iterative structural assembly simulations to generate 3D atomic models. The HP’s function is inferred from these 3D models by structurally matching them with known proteins. Phyre2, SWISS-MODEL, PS^2^, and I-TASSER all measure the quality of their resulting models though differences exist in how models are measured for quality. I-TASSER also provides functional annotations on ligand-binding (*i.e.*, active) sites, Gene Ontology terms, and Enzyme Commission numbers not provided by the other programs, though 3DLigandSite competes by providing active site characterization and ligand predictions for models produced by Phrye2. Further, potential binding partners for HPs can be predicted from programs separate from 3D modeling programs. For example, STRING (Snel et al., 2000; Szklarczyk et al., 2019) and STITCH (Kuhn et al., 2008; Szklarczyk et al., 2016) are databases of protein-protein and protein-chemical interactions, respectively. An HP’s function can be inferred from the network of proteins and chemicals identified from searching its amino acid sequence in the STRING and STITCH databases.

### Cellular Location Determination

Students finally consider the cellular environment in which their HP may exist. For classroom purposes, students focus on determining the cellular location of their HP using two programs, PSORTb and the SOSUI server. PSORTb consists of several analytical modules that each analyze one biological feature known to impact or be characteristic of a subcellular localization. PSORTb combines the results from each module to assess the likelihood of a protein being assigned a specific localization. Based on these likelihood assessments, a probability value between 0 and 10 for each of the five localization sites is determined. PSORTb considers 7.5 a good cutoff for assignment of a protein to a single cellular location (Yu et al., 2010). Similarly, SOSUI distinguishes between membrane and soluble proteins and predicts transmembrane helices in potential membrane proteins (Hirokawa et al., 1998; Mitaku and Hirokawa, 1999; Mitaku et al., 2002). To do this, SOSUI considers four physicochemical parameters (amphiphilicity index, hydropathy index, index of amino acid charges, and length of each sequence) to calculate grand averages of hydropathy (GRAVY). Positive GRAVY values indicate hydrophobic; negative values mean hydrophilic (Chang and Yang, 2013). For a more detailed analysis, ExPASy ProtParam can be used to calculate physicochemical parameters individually including aliphatic index, index of amino acid composition, length of each sequence, and GRAVY (Gasteiger et al., 2005; Artimo et al., 2012). ExPASy ProtParam also provides experimentally useful information such as instability index (*i.e.*, estimate of HP stability in a test tube), extinction coefficient (*i.e.*, measure of light absorbance at 280 nm wavelength), estimated half-life in mammalian reticulocytes, yeast, and *Escherichia coli*, and theoretical pI (*i.e.*, isoelectric point, pH where the HP is electrically neutral). While the ability to determine cellular location for an HP does not distinguish outdated annotation from true HPs, cellular location can support re-annotation conclusions for outdated HPs drawn from other results generated from the Hypothetical Protein Characterization Project.

## Example Hypothetical Protein Characterization Projects

The following section contains examples to demonstrate possible Hypothetical Protein Characterization Project results that might be encountered in educational settings. The examples presented here utilized FASTA-formatted amino acid sequences acquired from the NCBI Protein database (Coordinators, 2018). The UniProt knowledgebase (UniProt, 2019) was consulted to highlight differences between knowledgebases. For consistency across projects, the following program parameters were used: (1) Default program settings for all programs, (2) The most similar non-HP sequence was reported from BLASTP analysis, making it the most relevant description for potential re-annotation, (3) PSI-BLAST results were generated from three iterations of each sequence to capture similar sequences more extensively as no significant change resulted from running additional iterations, and (4) The least similar non-HP sequence resulting from PSI-BLAST analysis was reported. Data for these example projects were collected between March 15–23, 2020.

### AUH26_00140 Should Be Re-annotated as an ABC Transporter Permease

To find an example of an HP with outdated annotation, the sequence similarity to a protein with determined structure approach to select HPs was used. Since we previously used this approach to examine HPs related to major facilitator superfamily proteins related to antibiotic resistance in *S. aureus* (Marklevitz and Harris, 2016), we browsed the PDB for multidrug resistance transporters related to antimicrobial resistance. We performed PSI-BLAST on approximately five randomly selected transporters before finding a transporter with HPs, a process taking less than 30 min, demonstrating the feasibility of sequence similarity to a protein with determined structure approach to identify outdated HPs. We found PSI-BLAST of the multidrug ABC transporter Sav1866 from *S. aureus* (PDB accession: 2ONJ) identified HPs. We selected AUH26_00140 (96% query coverage, 38.89% identity, *E*-value = 6.0 × 10^–142^) over three other HPs with lesser similarity (W538_02582 from *S. aureus* VET0261R, W475_02351 from *S. aureus* VET0166R, and V089_02512 from *S. aureus* GD2010-115). We noted that AUH26_00140 was not included in the UniProt knowledgebase. The 592-amino acid sequence for AUH26_00140 is below:

>OLC18526.1 hypothetical protein AUH26_00140 [*Candidatus Rokubacteria* bacterium 13_1_40CM_69_96] MPLGPYRRLFVYLRPHVPVLVLGACLALIVSGMEGLTAWLV KPVMDDIFIRRDGLMLKLIPLALLAVYVVKGVARYLQSYLM AAVGERVVARLRRELYTHIQSMPLSFFSDVHSADLMSRILTD VTRLARLSSGVLVMGVRQLGTIAALLVVMLAREWALTLTA LVAFPAIALIVRTIGRRLYTINKRTQERVAQLAVLLHESFSGTK IVKAFGRERHEQARFDALNDRLLNLSLKNVRADEITEPLME IAGALGIMAVLWYGGYRVIEGHMTPGTLFSFTAAALMLYG PVRRLSRSLNVVQQSTASVERVFHILELPPAITDRPGATRLET FTRALAFERVDFRYGDADEMTLKEISLEIRKGEVVAFVGMS GAGKSTLMDLVPRFHDVTAGRITLDGRDLRDVTQASLRAQ LGVVTQETFLFSDTIRYNIAYGRPDATFEEIVRAARQAHAH DFTLACPDGYDTLVGERGVRLSGGQRQRIAIARAFLKNPPIL ILDEATSDLDAESEFMVQQALAELMHGRTVFVIAHRLATVR NADRIVVVHDGRIAEIGRHEELIARDGIYRRLYALQMEGFPG EQVGGPGGPLRPR

When AUH26_00140 was used as query for BLASTP, the most similar non-HP sequence was an ABC transporter permease from *Candidatus rokubacteria* bacterium (97% query coverage, 98.96% identity, *E*-value = 0.0), which is a strong indicator that AUH26_00140 has outdated annotation. PSI-BLAST results included mostly lipid A export permease protein MsbA (98% query coverage, *E*-value = 0.0, 49.06% identity) and no HPs, further supporting BLASTP results.

The NCBI Protein database did not list any domains. CD-Search identified COG1132 (*E*-value = 0.00), a domain that spans most of AUH26_00140 (amino acids 3 to 576) which is associated with the ATPase and permease component of the ABC-type multidrug transport system. Pfam also found two matches: (1) an ABC transporter transmembrane region (CL0241, *E*-value = 3.2 × 10^–52^) spanning amino acids 21 to 291, and an ABC transporter domain (CL0023, *E*-value = 3.3 × 10^–33^) that spans amino acids 354 to 503, supporting results identified by CD-Search.

**FIGURE 3:**
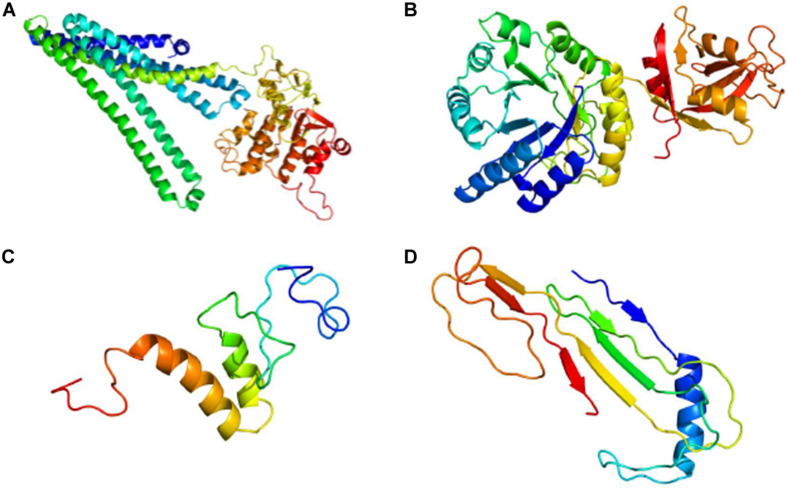
Predictive 3D Models for Hypothetical Protein Characterization Project Examples. **(A)** Completeness of Phyre2 model of AUH26_00140 shows AUH26_00140 has outdated annotation. **(B)** Completeness of Phyre2 model of L2624_01843 suggests L2624_01843 has outdated annotation. **(C)** Lack of completeness of Phyre2 model of WP_002214142 supports the conclusion that WP_002214142 is an example of indeterminate annotation. **(D)** Lack of completeness of Phyre2 model of YP_009724396 indicates YP_009724396 is an example of indeterminate annotation. All images are colored by rainbow from N terminus to C terminus.

Phyre2 generated a tertiary structure model for AUH26_00140 with 100% confidence from part of an X-ray diffraction structure of a heterodimeric ABC transporter from *Thermotoga maritima* (model template c3qf4A) whose protein sequence covered 96% of AUH26_00140’s sequence with 31% identity (Figure 3A). From this model, 3DLigandSite predicted a 14-amino acid binding site that could bind to adenosine triphosphate (ATP), adenosine diphosphate (ADP), and magnesium.

PSORTb predicted that AUH26_00140 is a cytoplasmic membrane protein (localization score = 10). These results are supported by SOSUI, which calculated AUH26_00140 to be a membrane protein (GRAVY = 0.168920) with five transmembrane helices. While additional analysis, such as comparison of physiochemical properties, multiple sequence alignment, and phylogenetic tree analysis, are needed to fully support re-annotation, these results here suggest AUH26_00140 likely has outdated annotation and should be re-labeled to be a ABC transporter permease in keeping with its closest similar sequence.

### L2624_01843 Should Be Re-annotated as a DUF871-Containing Outer Surface Protein

L2624_01843 from *Listeria monocytogenes* was originally characterized as part of student’s Hypothetical Protein Characterization Project using the student-directed approach for HP selection. NCBI Protein database listed L2624_01843 as an HP. L2624_01843 was not included in the UniProt knowledgebase. The 362-amino acid sequence for L2624_01843 is provided below:

>AKI46902.1 hypothetical protein L2624_01843 [*L. monocytogenes*] MRKLGISVFPQHVALEESL EYIETAAKYGFSRIFTCLISANDEAEFAKLETICKRAKELGFD VIADVDPTVFESLNITYKELDRFKELGLAGLRLDLGFSGSEE AAMSFDDTDLKIELNISNGTRYVENILSYQANVGNIIGCHN FYPRKYTGLSRKHFLRTSKQFKDLNLRTAAFVSSNSGEFGPW FVVDGGLPTMEEHRGVDITVQAKDLWNTGLIDDVIVGNM FASEDELRALSELNRNELQLAVEFLDGATDVEKEIVLTQKHF NRGDASEYVLRSTMTRVNFKQFDFPAHDTNTIAKGDVTID NDGYERYKGEMQVALQEMENSGNTNIVARIVPEERYLLDTI LPWQHFRLVEKKK

When L2624_01843 was used as query for BLASTP, all identified similar sequences had DUF871 domain-containing protein annotation (100% query coverage, 99.17% identity, *E*-value = 0.0). While most similar sequences identified by PSI-BLAST for L2624_01843 are DUF871 domain-containing proteins, a few sequences had outer surface protein descriptions with the closest sequence being EFR87458.1 which is found in *Listeria marthii* FSL S4-120 (100% query coverage, 98.90% identity, *E*-value = 0.0).

The NCBI Protein database showed that L2624_01843 contains a conserved COG3589 region that has an unknown function that spans 361 amino acids (99.7% of the protein). CD-Search showed COG3589 was similar (covering amino acids 1 to 361, *E*-value = 0.00) to the DUF871 domain superfamily, which was confirmed by Pfam that found DUF871 was the only significant match (covering amino acids 1 to 357, *E*-value = 3.1 × 10^–136^).

Next, the tertiary structure and potential ligand binding partners for L2624_01843 were predicted. Phyre2 generated a protein model for L2624_01843 with 100% confidence from the crystal structure of an outer surface protein from *Bacillus cereus* (model template c1x7fA, PDB accession 1X7F_A) whose protein sequence covered 95% of L2624_01843’s sequence with 51% identity (Figure 3B). Interestingly, according to NCBI’s Protein database, 1X7F_A is 385 amino acids long and contains a DUF871 domain spanning across amino acids 28 to 384. 3DLigandSite predicted a binding site involving 32 amino acids, mostly comprised of residues 176–185 and 222–228, that bound with the following heterogens: NADPH dihydro-nicotinamide-adenine-dinucleotide phosphate (NDP), flavin mononucleotide (FMN), magnesium, NADP nicotinamide-adenine-dinucleotide phosphate (NAP), zinc, b-D-mannose (BMA), a-D-mannose (MAN), and calcium.

SOSUI calculated L2624_01843 to be a soluble protein (GRAVY = −0.328453) with no transmembrane helices, which supported PSORTb predictions that L2624_01843 was a cytoplasmic protein (localization score = 7.50). We noted that PSORTb is unable to detect outer surface as a cellular location (Yu et al., 2010). Taken together, these data suggested that L2624_01843 should be re-labeled as a DUF871-containing Outer Surface Protein though experimental examination of DUF871 is needed to further refine L2624_01843’s annotation.

### WP_002214142 Is a True Hypothetical Protein

WP_002214142 from *Yersinia pestis* plasmid pMT1 was originally characterized as part of student’s Hypothetical Protein Characterization Project using the instructor-directed class pet microbe approach for HP selection. WP_002214142 was labeled as a hypothetical (*i.e.*, uncharacterized) protein in both NCBI Protein and UniProtKB databases. The 77-amino acid sequence is provided below:

>WP_002214142.1 MULTISPECIES: hypothetical protein [Bacteria] MAQAIPSTSVCSTKRTRPPMLVALNGH PVSRRLKTPTSYRQATEQPSDSLQATICRNRTLGRLMRVAIIK PTRKQIV

BLASTP identified several HPs from various species with similar sequences to WP_002214142. PSI-BLAST was not able to identify similar sequences for WP_002214142 that were not HPs and new sequences could not be detected above the 0.005 threshold from the second iteration of PSI-BLAST. In summary, no sequences from non-HPs were identified.

WP_002214142 contains no documented domains according to NCBI’s knowledgebase, either Protein database or the CDD. Pfam also could not detect any domains. Lack of identified domains is a good indication that the HP under characterization is a true HP.

Phyre2 generated a tertiary structure model for WP_002214142 with 31.8% confidence from part of an X-ray diffraction interferon-induced RNA binding protein from *Homo sapiens* (model template c6c6kD) whose protein sequence covered 30% of WP_002211802’s sequence with 52% identity (Figure 3C). Low model confidence and similarity to the template supports the conclusion that WP_002214142 is a true HP. To further support this conclusion, 3DLigandSite was unable to predict a binding site or ligand binding partners from this model.

SOSUI calculated WP_002214142 to be a soluble protein (GRAVY = −0.425), though PSORTb could not predict a cellular location for WP_002214142 (localization score = 2.00). Project results taken together do not provide sufficient evidence to re-label WP_002214142 in public knowledgebases. Therefore, experimental examination is needed before WP_002214142’s annotation can be improved.

### ORF8 (YP_009724396.1) Is a Viral Example of a True Hypothetical Protein

While the Hypothetical Protein Characterization Project was optimized for use on bacterial species, students frequently want to apply it to other organisms. A virus that students have recently want to use for their projects is Severe Acute Respiratory Syndrome coronavirus 2 (*i.e.*, SARS-CoV-2), the causative agent of COVID-19 (Wang et al., 2020). So, for this example, ORF8 (*i.e.*, ORF8) was randomly selected from the SARS-CoV-2 genome. When this example was prepared, ORF8 was labeled as an HP in the NCBI Protein database and not found in UniProt. The 121-amino acid sequence is provided below:

>YP_009724396.1 ORF8 protein [Severe acute respiratory syndrome coronavirus 2] MKFLVFLGIITTVAAFHQE CSLQSCTQHQPYVVDDPCPIHFYSKWYIRVGARKSAPLIELC VDEAGSKSPIQYIDIGNYTVSCLPFTINCQEPKLGSLVVRCSF YEDFLEYHDVRVVLDFI

All but one protein identified by BLASTP had ORF8 annotation and came from SARS-CoV-2. The one sequence that was not an ORF8 was a HP from Bat SARS-like coronavirus (100% query coverage, 94.21% identity, *E*-value = 8 × 10^–81^). Most similar sequences identified by PSI-BLAST for ORF8 were also HPs or proteins with vague descriptions (*e.g.*, ORF8a or ORF10). However, one sequence (AAP51236.1), which came from Human SARS coronavirus (SARS Co-V) GD01, had a BGI-PUP(GZ29-nt-Ins) description (98% query coverage, 29.03% identity, *E*-value = 4 × 10^–42^). The BGI-PUP(GZ29-nt-Ins) description is associated with a SARS-CoV isolate with a 29 nucleotide insertion at the relative position 27,995 in its genome (Pavlovic-Lazetic et al., 2005).

The NCBI Protein database listed no domains for ORF8. However, CD-Search showed a functionally uncharacterized corona_NS8 superfamily domain conserved in coronaviruses (100% query coverage, *E-*value = 1.87 × 10^–39^). CD-Search results were confirmed by Pfam that found Coronavirus NS8 protein was the only significant match (*E*-value = 3.8 × 10^–44^). Both CD-Search and Pfam aligned the corona_NS8 superfamily domain to residues 1 to 118 in ORF8.

To predict the tertiary structure for ORF8, Phyre2 generated a protein model for ORF8 with 33.3% confidence from the immunoglobulin-like beta-sandwich fold of an X-ray diffraction of the ORF7a accessory protein from SARS-CoV (model template d1xaka) whose protein sequence covered 17% of ORF8’s sequence with 30% identity (Figure 3D). From this limited model, 3DLigandSite was unable to predict potential binding site or ligand binding partners.

With regards to cellular location, SOSUI calculated ORF8 as a soluble protein (GRAVY = 0.219). PSORTb could not predict a cellular location for ORF8 because PSORTb cannot analyze viral sequences. Taken together, these data suggested that more experimental examination is needed before ORF8’s annotation can be improved, which is not surprising given the novelty of SARS-CoV-2 at this time.

## Discussion

The Hypothetical Protein Characterization Project is a valuable educational tool where students learn and apply knowledge of computational programs that can assist with ongoing manual curation efforts to improve genome annotation (Figure 1). This project incorporates interdisciplinary concepts to identify and predict HP characteristics, such as sequence similarities, domains, 3D structure, ligand binding partners, and cellular location. Project results are used to determine whether an HP has outdated or indeterminate annotation. Individual and collective results from student projects can be used to improve public database annotation. While current NCBI knowledgebase protocols dictate that only the research group that deposited the genome can change its annotation, depositor contact information is usually provided. While contact information may need to be updated, students are encouraged to use internet search resources to find and share their HPCP results for outdated HPs with the genome’s depositor(s). This provides students with an opportunity to establish and develop professional connections that could benefit them throughout their careers. Further, individual and collective results from student projects are often welcomed for scientific conference poster presentations, which further stimulates student motivation, learning opportunities, and ideally scientific employability.

The project is versatile and customizable to accommodate a wide variety of learning objectives. The project can be used in both online and in-seat educational settings for undergraduate and graduate classes in microbiology, bioinformatics, genetics, and/or biochemistry. HP analysis objectives and programs can be modified based on the instructor’s learning objectives, and we recommend instructors test programs immediately prior to classroom use to ensure functionality as programs are often temporarily taken off-line for maintenance and updates. Further, this project can be expanded through advanced approaches to HP selection, such as differential gene expression or phylogenetic relations, and additional HP analysis to provide an advanced, research-oriented project that is well suited for undergraduate capstone, honor’s, and experiential learning projects as well as Master level theses (Table 2). Given the variety of potential HP selection approaches and programs for HP analysis, students and instructors are encouraged to find, develop, and/or use these and other methods of selecting and analyzing HPs to best suit their specific needs.

Further, the project was designed to stimulate classroom discussion based on the methodology and interpretation of variations in results from different knowledgebases and HP analysis programs (Table 3). Classroom discussion can begin with comparing and contrasting information found on the HP between NCBI Protein database (Coordinators, 2018) and UniProt knowledgebase (UniProt, 2019). As seen from examples provided in this paper, in some cases like WP_002214142, HP information provided is the same between Protein and UniProt. In other cases, like AUH26_00140 there are differences in HP inclusion and/or provided information. Similar discussions that compare analysis programs can be applied to each objective. For example, if an instructor wants to examine program methodology differences, students can discuss why results first iteration PSI-BLAST results are the same as BLASTP results and how PSI-BLAST uses BLASTP results to identify distant similar sequences. An instructor that wants to continue discussing impacts of knowledgebase inclusion could similarly emphasize program inclusion by discussing similarities and differences in methodology and generated results between Pfam and CDD, which includes a number of external source databases including Pfam (Marchler-Bauer et al., 2017; Lu et al., 2020). Instructors may decide to have students explore other bioinformatic resources to supplement or replace analysis databases and programs described in this paper to stimulate student discussion. Finally, though we used default settings for our examples here, student discussion can be generated around how and why variations from default settings change results of program analysis. Taken together, this discussion highlights the educational aptitude of the Hypothetical Protein Characterization Project.

### Random Selection of Hypothetical Proteins Is Best for Classroom Use

Random selection of HPs for the Hypothetical Protein Characterization Project is optimal for beginning students with no prior experience in bioinformatics or statistics (Table 2). Random selection is the easiest HP selection method since it does not require extra computational analysis. This makes random selection of HPs good for undergraduate classroom use, particularly as a multi-step individual assignment. Example assignment instructions with grading rubrics and their 15-week course schedule designed for use in student-directed random HP selection are included in Supplementary Materials.

Giving students complete autonomy in HP selection (*i.e.*, student-directed) empowers them to take ownership of their projects. Students will naturally select HPs from a wide range of species, the student-directed approach is good for identifying both outdated and true HPs that can be used as examples in large-class discussions. However, programs can vary in their ability to generate accurate results from diverse species. For example, PSORTb requires its users to provide the type of microbe (*i.e.*, Gram-negative or Gram-positive) that the amino acid sequence came from. If the student selects an HP from a *Mycobacterium* that has an advanced cell wall, PSORTb may struggle to provide clear and accurate results. Further, PSORTb was not designed to analyze eukaryotic HPs, though its complementary program WoLF PSORT can analyze eukaryotic HPs (Horton et al., 2007), which can cause confusion and frustration among students and instructors alike if the student selects a eukaryotic protein for study. To avoid such complications, we recommend some instructor-imposed limitations in HP selection (*i.e.*, instructor-directed) for classroom use. Partially instructor-directed approaches, such as the class pet microbe discussed earlier, are better than the instructor simply assigning HPs to students directly (*i.e.*, completely instructor-directed) as this approach allows students to retain some autonomy in the selection process while still reducing the confusion that can result from interpreting results across diverse species. However, both partial and complete instructor-directed HP selection approaches may not generate ample examples of outdated HPs needed for large-class discussions unless the instructor is careful to select HPs from older genomes that are more likely to have outdated annotation compared to recently published genomes.

### Hypothetical Protein Selection via Differential Gene Expression Is Best for Advanced Students With the Ability to Conduct Laboratory Experimentation

Selecting HPs based on differential gene expression is a great approach that expands the Hypothetical Protein Characterization Project by incorporating statistical analysis of gene expression data to identify HPs that have a specific biological relevance. Analysis of gene expression differences adds more scientific rationale to the project, which makes true HPs identified by the project using the differential gene expression approach potentially valuable in addressing serious biological questions, allowing a priority to be placed on their experimental examination. While the differential gene expression approach can be used in upper-level undergraduate and graduate classrooms where statistics is a pre-requisite, without laboratory access students cannot fully realize their educational potential (Table 2). For this, advanced educational applications such as first-year experiential learning courses, undergraduate honor’s and capstone projects, or graduate work where students have access to laboratory resources to experimentally examine true HPs identified from this approach are needed. Further, having a laboratory component to the project can be helpful if the instructor wants to share student project results within the broader biological sciences community.

This paper discussed three progressively more challenging ways to identify HPs using differential gene expression. Single-gene analysis, the easiest way to use differential gene expression to identify HPs, requires an understanding of statistics since it uses statistical methods such as a Student’s *T*-test to select HPs through via differential gene expression. Singular enrichment analysis improves upon single-gene analysis by selecting overlapping HPs between differential expression comparisons so that HPs can be grouped based on their potential biological relevance. However, due to its dependence on single-gene analysis for HP selection, singular enrichment analysis only considers HPs that meet a specific statistical cut-off, producing long lists of differentially expressed HPs that may contain redundancy. To overcome these limitations, GSEA considers all genes during analysis by removing the need for a statistical cut-off (Tipney and Hunter, 2010). GSEA is extremely complex, and best for advanced educational projects such as a Master thesis, where the goal is to identify true HPs whose immediate experimental examination could directly enhance scientific understanding of a variety of biological mechanisms (Goad and Harris, 2018).

### Further Computational Analysis Expands the HPCP for Advanced Students Without Laboratory Access

As mentioned earlier, selection of HPs via sequence similarity to a protein with determined structure is inherently useful for finding outdated HPs that do not require further experimental examination (Marklevitz and Harris, 2016). Results generated from HPs selected by this approach become supporting evidence toward the conclusion that the selected HPs should be re-annotated in keeping with similar sequences with established annotation. Due to this, 3D predictive models generated from this project, like the one we provided for AUH26_00140, should be further validated for accuracy. Procheck and other free web-based programs check the stereochemical quality of a model’s structure, such as deviations from ideal bonding angles and bond length, and produce a Ramachandran plot identifying outliers and clashing contacts which is a standard part of structure analysis before deposition (Praznikar et al., 2019). Further, after completion of the project, selected HPs and identified similarly sequenced proteins with established annotation should undergo additional comparisons to support re-annotation conclusions. Examples of additional computational analyses include multiple sequence alignment, physiochemical properties, and phylogeny tree builder, performed by programs such as PROMALS3D (Pei et al., 2008) or CLUSTAL Omega (Thompson et al., 1994; Madeira et al., 2019), ExPASy ProtParam (Artimo et al., 2012), and the PHYLIP suite (Lim and Zhang, 1999; Retief, 2000; Abdennadher and Boesch, 2007), respectively. These additional analyses make the phylogenetic relations approach for selecting HPs a complete bioinformatics project that is ideal for undergraduate honor’s and capstone projects or as part of graduate work where scientific rationale for the study is needed but students lack access to a laboratory for further experimental examination.

### Knowledgebases Are Constantly Improving

The overall goal of the Hypothetical Protein Characterization Project from a student perspective is to assist in improving genome annotation. To emphasize the speed at which knowledgebases update as well as the importance of improving genome annotation, we re-ran the project on ORF8 on June 10, 2020, to see how results may have changed in a short time under substantial pressure to computationally and experimentally characterize SARS-CoV2 due to the COVID-19 pandemic. We found that NCBI Protein database updated the protein’s description in the public record from HP to ORF8 protein (Severe acute respiratory syndrome coronavirus 2). The record now shows a corona_NS8 domain for ORF8 where it was not listed in March despite previous CDD and Pfam identification. In March, CDD and Pfam described the corona_NS8 domain as a functionally uncharacterized superfamily domain conserved in coronaviruses. While the statistical values have not changed, now the description details a superfamily of immunoglobulin (Ig) domain proteins without mention of anything still being uncharacterized. While UniProt did not have an entry for ORF8 in March and still does not have one using the same identifies as NCBI, UniProt has now added ORF8 as a 121 amino acid long, non-structural protein 8 under the identifier P0DTC8 (NS8_SARS2). We used the WayBack Machine web archival site^2^ to confirm P0DTC8 did not exist in UniProt in March. 3D predictive modeling and cellular location results did not change between March and June, though we expect modeling for ORF8 to improve when the structure of ORF8 or one of its homologs has been elucidated.

Given the high number of newly sequenced genomes deposited regularly to public knowledgebases, there will be plenty of HPs for use in the Hypothetical Protein Characterization Project for years to come. Further, proteins with vague annotation descriptions (*e.g.*, membrane protein) and no gene symbol may also benefit from characterization using this project. The quick update in the annotation of ORF8 due to the COVID-19 pandemic highlights how manual review can improve genome annotation when ample resources are available. This paper provides a tool that turns students into manual reviewers of genome annotation while learning valuable interdisciplinary concepts. Application of the Hypothetical Protein Characterization Project in educational settings worldwide has the potential to significantly improve public knowledgebases and the scientific conclusions derived from their information.

## Data Availability Statement

The datasets presented in this study can be found in online repositories. The names of the repository/repositories and accession number(s) can be found below: https://www.ncbi.nlm.nih.gov/, YP_009724396.1; https://www.ncbi.nlm.nih.gov/, WP_002214142; https://www.ncbi.nlm.nih.gov/, AKI46902.1; https://www.ncbi.nlm.nih.gov/, OLC18526.1.

## Author Contributions

LH conceived the presented idea, developed the theory, and performed the computations. ZA verified the computations and manuscript citations. LH took the lead in writing the manuscript in consultation with SG. All authors contributed to the article and approved the submitted version.

## Conflict of Interest

The authors declare that the research was conducted in the absence of any commercial or financial relationships that could be construed as a potential conflict of interest.

## Footnotes

1 https://www.youtube.com/channel/UCEE6oecA8YKQip9VaqOOHbg

2 https://archive.org/web/
