# Supplementary material for: An Educational Bioinformatics Project to Improve Genome Annotation
Source: Front Microbiol. 2020 Dec 7;11:577497. doi: 10.3389/fmicb.2020.577497 (PMC7750189; doi:10.3389/fmicb.2020.577497)
Supplement: Supplementary file 1 [file Data_Sheet_1.docx]

Supplementary Material

# Example Syllabus for a Bioinformatics Undergraduate Class Using the Hypothetical Protein Characterization Project

| **Week** | **Topic** |
| --- | --- |
| **1** | Introduction and databases |
| **2** | Genomic assembly and gene prediction |
| **3** | Genomic assembly and gene prediction |
| **4** | Genomic sequences and alignment |
| **5** | Multiple sequence alignment |
| **6** | Protein motifs and domains |
| **7** | **Hypothetical Protein Characterization Project Pre-draft DUE**  Protein tertiary structure prediction  Protein-protein interactions |
| **8** | Cellular location |
| **9** | **Hypothetical Protein Characterization Project DUE**  Systems biology |
| **10** | Molecular phylogeny and evolution |
| **11** | Gene expression and regulation |
| **12** | Gene set enrichment and clustering analyses |
| **13** | tRNA detection |
| **14** | Human variation and disease |
| **15** | **Final Exam** |

# Hypothetical Protein Characterization Project Pre-draft Assignment Instructions

Please refer to the Hypothetical Protein Characterization Project instructions for project details. Briefly discuss what BLASTP, PSI-BLAST, Pfam, and CD-Search are used for and how they work (*i.e.*, get their results). Provide information to locate your chosen sequence (*i.e.*, link with locus tag number). Show results from each of 4 tests listed above for your chosen sequence. These will become part of your final Hypothetical Protein Characterization Project paper.

# Hypothetical Protein Characterization Project Pre-draft Assignment Grading Rubric

Programs to evaluate: BLASTP, PSI-BLAST, Pfam, CD-Search

Descriptions of each program (4 programs @ 5 points): 20 points

Results from each program (4 programs @ 5 points): 20 points

Inclusion of chosen sequence information: 5 points

Grammar, spelling, APA format: 5 points

Total: 50 points

# Hypothetical Protein Characterization Project Assignment Instructions Using Student-directed Random Selection with a Protein Size Limitation

Select 2 hypothetical proteins from NCBI that is over 200 amino acids in size. Note annotations and information for your proteins from both NCBI and UniProt and include this information in your paper. Use the following programs and the steps learned in class to determine the possible function of your proteins:

1. Sequence Homology (BLASTP, PSI-BLAST)

2. Domain Identification (Pfam, CDD)

3. Protein Structure (Phyre2)

4. Ligand Binding (3Dligand)

5. Cellular Location (PSORTb, SOSUI)

Write a scientific paper on your findings. In the introduction, discuss why you selected your species, strain, and proteins. Perform a literature search and briefly discuss what background information on identification of proteins you found. Conclude the introduction with a hypothesis or thesis statement. Discuss programs used for each analysis including how the programs accomplish their tasks in a Materials and Methods section. Discuss acquired results in a Results section. Include tables and figures to show all raw data generated from the examinations above as needed. Discuss their meanings in a separate Discussions section. If there were discrepancies among results generated, discuss potential reasons why. Also, discuss if the literature supports those meanings. End the paper with a conclusion paragraph that summarizes the hypothesis and main findings of the project. The paper must have APA format, references, and in-text citations with proper grammar and spelling. There are no word requirements on the paper. Please be concise yet detailed and thorough.

# Hypothetical Protein Characterization Project Assignment Instructions

| **Criteria** | **10-8** | **7-4** | **3-0** |
| --- | --- | --- | --- |
| Introduction | Detailed background provided & the purpose or hypothesis is clearly stated | Some background provided and purpose or hypothesis is stated | Very little info provided, or info is inadequate, purpose or hypothesis may not be stated |
| Sequence Homology | Methods were detailed & results included raw data and discussion on what it meant | Some detail on methods & results, some raw data & discussion on what it meant | Very little info provided, or info is inadequate |
| Domain Identification |  |  |  |
| Protein Structure |  |  |  |
| Ligand Binding |  |  |  |
| Cellular Location |  |  |  |
| Discussion | Hypothesis is supported or rejected, relevance of procedure is explained, suggestions for further research included | Most parts of the discussion are presented adequately | Many parts of the discussion are missing or inadequate |
| APA format | No formatting errors per APA guidelines | Few errors in formatting per APA guidelines | Several errors in formatting per APA guidelines |
| References | References and in-text citations correctly cited | Errors in APA citation | No references cited |
| Grammar and spelling | No grammar and/or spelling errors | Few errors in grammar and/or spelling | Several errors in grammar and/or spelling |
